# Knowledge of Thrombolytic Therapy for Acute Ischemic Stroke among Community Residents in Western Urban China

**DOI:** 10.1371/journal.pone.0107892

**Published:** 2014-09-15

**Authors:** Juan Yang, Min Zheng, Shuqun Chen, Shu Ou, Jie Zhang, Ni Wang, Yingying Cao, Jian Wang

**Affiliations:** 1 Department of Neurology, the Second People’s Hospital of Chengdu, Chengdu, China; 2 Basic Medical College, Chongqing Medical University, Chongqing, China; 3 Department of Preventive Medicine, Chongqing Medical University, Chongqing, China; 4 The Second Affiliated Hospital Chongqing Medical University, Chongqing, China; Indian Institute of Integrative Medicine, India

## Abstract

**Background and Purpose:**

Thrombolytic therapy rate for acute ischemic stroke remains low, and improving public awareness of thrombolytic therapy may be helpful to reduce delay and increase chances of thrombolytic therapy. Our purpose was to survey the level of knowledge about thrombolytic therapy for acute ischemic stroke among community residents in Yuzhong district, Chongqing, China.

**Methods:**

In 2011, a population-based face-to-face interview survey was conducted in Yuzhong district, Chongqing. A total of 1500 potential participants aged ≥18 years old were selected using a multi-stage sampling method.

**Results:**

A total of 1101 participants completed the survey. Only 23.3% (95% *CI* = 20.8 to 25.8) were aware of thrombolytic therapy for acute ischemic stroke, of whom 59.9% (95% *CI* = 53.9 to 65.9) knew the time window. Awareness of thrombolytic therapy was higher among young people, those with higher levels of education and household income, those with health insurance, and those who knew all 5 stroke warning signs, while awareness of the time window was higher among those aged 75 years or older. Multivariate logistic regression analysis showed that awareness of thrombolytic therapy was independently associated with age, education level, health insurance and knowledge of stroke warning signs (*P*<0.05).

**Conclusions:**

In this population-based survey the community residents have poor awareness of thrombolytic therapy for acute ischemic stroke.

## Introduction

Recombinant tissue plasminogen activator (rt-PA) has been proven to be the most effective therapy for ischemic stroke [1]. However, less than 5% of acute ischemic stroke patients received thrombolytic therapy in China and other countries [2]–[5]. Various studies have shown that pre-hospital delay is the single most important reason for why patients with acute stroke do not receive rt-PA treatment [4]–[6]. Activating emergency medical services (EMS) should be the first and only response to suspected stroke symptoms, however, activation of EMS involves a complex “knowledge-to-action” process that requires adequate knowledge of stroke warning signs, proper recognition and interpretation of warning signs, and awareness of the need to activate EMS [7]–[9]. Some studies have found that knowledge of specific stroke warning signs resulted in increases in the odds of activate EMS, but the association between knowledge and the intention to activate EMS was weak in population-based studies [8]–[13] or in clinical patients with stroke symptoms [14]–[18]. Actually, some studies have showed that public awareness of stroke related knowledge has increased in recent years, but delays in stroke treatment remain prevalent, which indicates increasing public awareness of stroke warning signs may be insufficient to reduce pre-hospital delay [19]–[22].

There are a few studies that have found the low level of public knowledge about treatment for acute stroke, especially rt-PA [23]–[26]. The availability of rt-PA maybe the most important reason for stroke patients to arrive to the hospital as early as possible [23], therefore, awareness of thrombolytic therapy for acute ischemic stroke, particularly its time window, may lead to earlier activation of EMS, and reduce pre-hospital delay. So far there is no similar research report in China. We chose to determine the level of knowledge about thrombolytic therapy for acute ischemic stroke among community residents in Yuzhong district, Chongqing, China.

## Methods

### Sampling and Survey content

This is a cross-sectional study conducted in Yuzhong district of Chongqing, which is located in western China. The urban area of Chongqing municipality includes nine districts and Yuzhong district is located in the center area. It is the most developed and densest area in Chongqing, there are 12 blocks and about 660,000 permanent population in this area. According to a recent investigation report about cause of death, cerebrovascular disease is the third most common cause of death in Yuzhong district [27].

In this survey, 1500 households in Yuzhong district were randomly selected between March 20 and August 23, 2011. The study was approved by the ethics committees of the second affiliated hospital, Chongqing medical university. A detailed description of the epidemiologic methods employed for this study has been described previously [28]–[29]. Briefly, this study contained 4 sections: (1) Respondents’ demographic details such as sex, age, ethnicity, educational level, monthly household income, and health insurance. (2) The following two questions on knowledge of thrombolytic therapy were asked: “What kind of drug should be used to treat someone who is suffering from cerebral infarction: (i) traditional Chinese medicine for activating circulation to remove blood stasis, (ii) antibiotics, (iii) corticosteroids, (iv) thrombolytic agent, (v) other drugs”. Only one choice was allowed, and those who chose thrombolytic agent were asked, “Within how much time after a stroke begins this drug needs to be given to be effective?” (3) Awareness of stroke warning signs, respondents were asked to “Please judge whether the following 5 sudden symptoms are stroke symptoms”: (i) sudden difficulty in speaking, understanding, or slurred speech, (ii) sudden blurred vision in one or both eyes, (iii) sudden severe headache with unknown cause, (iv) sudden dizziness, difficulty in walking, loss of balance or co-ordination, (v) sudden numbness or weakness of the face and/or limb(s) on one side of the body. Respondents answered with “Yes”, “No”, or “Do not know/Not sure” for each listed symptom. (4) Sources of information about stroke knowledge, respondents were asked to “What are your sources of stroke related knowledge? (i) television, (ii) internet, (iii) newspaper, (iv) health magazine, (v) medical staff, (vi) others (family/friends)”. For this question, multiple answers were allowed.

### Data collection

The interview team made appointment with the selected household about the time and place before survey. After signing the informed consent form, the uniformly trained investigators conducted the survey through face-to-face interview with selected household member at the scheduled time and place. All the data were anonymized.

### Statistical analysis

All statistical analyses were completed using SPSS11.5 statistical software. Descriptive statistical analysis was employed to assess respondents’ general characteristics. The chi-square test was utilized to analyze the univariate relationship between demographic characteristics, awareness of stroke warning signs and knowledge of thrombolytic therapy. Multiple logistic regression analysis was used to identify factors independently associated with knowledge of thrombolytic therapy. *P*<0.05 was regarded as statistically significant.

## Results

A total of 1,101 respondents completed the questionnaires, with the response rate of 73.4%, included 419 men and 682 women. The mean age was 57.9±15.4 years (range, 18 to 91 years).

### Awareness of thrombolytic therapy for acute ischemic stroke

Among all of the respondents, 23.3% chose thrombolytic agent for the treatment of acute ischemic stroke, 42.0% chose traditional Chinese medicine. Univariate analysis showed that the awareness of thrombolytic therapy was unrelated to gender and ethnicity (*P*>0.05). The proportion awareness of thrombolytic therapy decreased with age, but higher awareness was among those with higher education level and household income. The awareness was also significantly higher among those with health insurance. In addition, awareness of thrombolytic therapy was significantly associated with the knowledge of stroke warning signs. The proportion awareness of thrombolytic therapy increased with the number of identification of stroke warning signs (*P*<0.001). However, even among those who could identify all 5 warning signs, only 34.9% chose thrombolytic agent for the treatment of acute ischemic stroke. Multiple logistic regression analysis showed that age, education level, health insurance and knowledge of stroke warning signs were independently associated with awareness of thrombolytic therapy (Table 1).

**Table 1:** Respondents’ awareness of thrombolytic therapy for acute ischemic stroke.

	Proportion (%)	95% CI	*P* *	AOR (95% CI)†
Total	23.3	20.8–25.8		
Gender			0.577	
Male	22.4	18.4–26.4		0.85 (0.62–1.17)
Female	23.9	20.7–27.1		1.00
Age Group (years)			<0.001	
18–36	40.9	31.9–49.9		3.68 (1.74–7.79)
37–55	24.7	19.8–29.5		2.96 (1.54–5.69)
56–74	23.5	19.8–27.1		2.60 (1.41–4.81)
≥75	8.4	4.2–12.7		1.00
Ethnicity			0.342	
Han	23.2	20.7–25.7		0.68 (0.25–1.83)
Other	31.8	12.4–51.3		1.00
Educational Level			<0.001	
Primary School or Below	10.5	7.1–14.0		1.00
Middle School	19.6	15.3–23.8		1.31 (0.80–2.14)
High School	26.4	21.1–31.7		1.71 (1.03–2.85)
College or Above	45.6	38.6–52.6		4.10 (2.35–7.15)
Monthly Household Income (yuan‡)			<0.001	
<2000	15.3	11.5–19.0		0.75 (0.43–1.33)
2000–3999	27.6	23.3–31.8		1.16 (0.69–1.96)
4000–7999	24.2	18.5–29.9		0.70 (0.40–1.22)
≥8000	31.7	22.8–40.7		1.00
Health Insurance			0.001	
Yes	25.3	22.5–28.1		1.81 (1.08–3.03)
No	13.4	8.4–18.4		1.00
Number of Knowledge of Stroke Warning Signs			<0.001	
0	9.9	5.0–14.8		1.00
1	11.7	5.9–17.4		1.24 (0.54–2.81)
2	15.2	10.2–20.1		1.49 (0.74–3.01)
3	28.4	23.0–33.8		3.02 (1.59–5.75)
4	31.3	24.9–37.8		3.34 (1.72–6.48)
5	34.9	27.8–42.0		3.70 (1.90–7.22)

* based on a chi-squared test.

† based on multiple logistic regression analysis.

‡ exchange rate: 100 dollars = 638 yuan.

### Awareness of time window for thrombolytic therapy

Of those who chose thrombolytic agent, 59.9% exactly answered the time window of thrombolytic therapy (within 3 hours after stroke onset), 20.2% answered the time window of more than 3 hours (from 4 hours to 15 days), and 19.8% reported they did not know (Table 2). Univariate statistical analysis showed that awareness of time window was not associated with gender, age, ethnicity, educational level, household income, health insurance and knowledge of stroke warning signs, though awareness of time window was higher among those aged 75 years or older than other age groups, and it also increased with education level and knowledge of stroke warning signs. However, multiple logistic regression analysis showed that the adjusted odds of awareness of time window were 5 times higher among those who could identified all 5 stroke warning signs, compared with those who did not identified anyone of stroke warning signs (adjusted odds ratio [AOR], 5.43; 95% Confidence Interval [CI], 1.46 to 20.16).

**Table 2:** Respondents’ awareness of treatment time window among those aware of thrombolytic therapy.

	Proportion (%)	95% CI	*P* *	AOR (95% CI)†
Total	59.9	53.9–65.9		
Gender			0.217	
Male	64.9	55.2–74.5		1.61 (0.91–2.84)
Female	57.1	49.5–64.7		1.00
Age Group (years)			0.754	
18–36	57.4	43.3–71.6		0.95 (0.22–4.15)
37–55	62.2	51.1–73.2		1.05 (0.27–4.15)
56–74	58.2	49.4–66.9		0.69 (0.19–2.52)
≥75	71.4	47.8–95.1		1.00
Ethnicity			0.587	
Han	60.4	54.3–66.5		2.56 (0.48–13.69)
Other	42.9	6.2–79.5		1.00
Educational Level			0.882	
Primary School or Below	56.3	39.1–73.4		1.00
Middle School	57.6	45.7–69.5		1.05 (0.42–2.65)
High School	60.0	48.5–71.5		0.84 (0.33–2.15)
College or Above	62.9	52.9–73.0		0.83 (0.31–2.23)
Monthly Household Income (yuan‡)			0.052	
<2000	48.1	34.8–61.5		0.43 (0.16–1.14)
2000–3999	57.6	48.7–66.5		0.64 (0.27–1.53)
4000–7999	73.1	61.0–85.1		1.47 (0.54–3.99)
≥8000	66.7	50.6–82.8		1.00
Health Insurance			0.055	
Yes	61.8	55.6–68.0		2.35 (0.89–6.24)
No	41.7	21.9–61.4		1.00
Number of Knowledge of Stroke Warning Signs			0.248	
0	35.7	10.6–60.8		1.00
1	50.0	23.8–76.2		2.18 (0.43–11.12)
2	53.3	35.5–71.2		1.91 (0.48–7.68)
3	59.2	48.2–70.3		2.76 (0.79–9.63)
4	63.5	51.6–75.4		3.05 (0.85–10.92)
5	68.3	56.6–80.1		5.43 (1.46–20.16)

* based on a chi-squared test.

† based on multiple logistic regression analysis.

‡ exchange rate: 100 dollars = 638 yuan.

The proportion awareness of thrombolytic related knowledge including thrombolytic therapy and time window increased with knowledge of stroke warning signs. However, even among those who could recognize all 5 warning signs, only 23.8% chose thrombolytic agent and knew the time window. In addition, among those who could identify all 5 warning signs and chose thrombolytic agent, 31.7% did not know the time window of 3 hours.

### Sources of acquiring stroke related knowledge

In the study, the proportion of respondents who chose family/friends, television and newspaper as the main sources of information about stroke was 40.6%, 32.2% and 18.1% respectively. However, only 16.7% of respondents selected medical staff.

## Discussion

This population-based study found that about 1/5 of respondents were aware of thrombolytic therapy for acute ischemic stroke, and nearly one half of them could correctly answer the treatment time window of 3 hours, thus, only 1/10 of the respondents were aware of thrombolytic therapy as well as time window. Compared with the results from surveys in other countries, our community residents’ awareness of thrombolytic therapy as a treatment for acute ischemic stroke was very poor. The survey of Morgenstern et al. [24] found that 48% Mexican Americans and 57% non-Hispanic whites were aware of thrombolytic therapy for ischemic stroke. Ferris et al. [25] conducted a survey of US women and found that 92% of whites, 84% of blacks and 79% of Hispanics were aware of thrombolytic therapy for acute ischemic stroke. Anderson et al. [23] reported that 32.3% respondents were aware of the existence of rt-PA treatment for acute stroke, of whom 52.7% knew it needed to be administered within 3 hours of symptoms onset. In 2005, one population-based survey in Cincinnati, Ohio area estimated that only 19% were aware of rt-PA [26]. In addition to the cultural settings and racial disparities, differences in question structure and format may be the most important reason for the different results of the surveys aforementioned. For example, the survey of US women used a yes/no question format [25], and the Cincinnati survey used an open-ended question that directly assessed awareness of rt-PA [26], whereas our study adopted a closed-ended question to evaluate the awareness of the early treatment measures for acute ischemic stroke. Interestingly, almost half of the respondents selected traditional Chinese medicine for activating circulation to remove blood stasis as the treatment measures for acute ischemic stroke, which suggested that traditional Chinese medicinal therapy has a profound influence on Chinese people.

In this survey, we found that the level of knowledge about thrombolytic therapy was associated with socio-demographic characteristics, including age, education level, household income, and health insurance. Awareness of thrombolytic therapy was higher among those youth aged 18–36 years old than other age groups. The proportion awareness increased with educational level and household income and was higher among the respondents with health insurance, but it was not associated with gender or ethnicity. However, one similar survey by Anderson et al. [23] showed that awareness of rt-PA was higher among middle aged adults, females, whites, and those with higher education and income. Otherwise, we found that respondents’ thrombolytic related knowledge increased with the knowledge of stroke warning signs, however, even among those who could recognize all 5 stroke warning signs, only about 1/3 chose thrombolytic agent, and about 1/5 not only chose thrombolytic therapy but also knew the time window. In addition, among those respondents who could recognize all 5 warning signs and chose thrombolytic agent for the treatment of acute ischemic stroke, almost 1/3 did not know the time window. Our results and the similar results from other reports [23],[26] demonstrated that the level of public knowledge about thrombolytic therapy was low, even among those with adequate knowledge of stroke warning signs, which might be another important factor for failing to active EMS timely and delay for stroke treatment.

The results we found in this study indicated that public knowledge about thrombolytic therapy is relatively low and needs to be improved. Previous stroke public education campaigns mainly focus on improving public recognition of stroke symptoms, which have been showed to be effective in raising awareness of stroke warning signs and reduce delay in presenting to the emergency department [30]–[33], but have limited impact on behavior [34]. In fact, activation of EMS immediately after witnessing a stroke symptom requires not only adequate knowledge of stroke symptoms but also the belief that use of EMS will result in better outcomes [9]. Awareness of thrombolytic therapy may increase motivation of rapid activating EMS, because thrombolytic therapy is the only effective treatment for acute ischemic stroke. Therefore, future public education should emphasize increasing the public knowledge about acute stroke treatment especially thrombolytic therapy and its time window, in addition to awareness of stroke warning signs.

In the present study, about half of respondents chose mass media including television and newspaper as the information source about stroke. This result is similar to another Chinese study which showed that the main sources of information about stroke were television (74.4%), doctors (63.2%) and newspapers (61.8%) [35]. However, the proportion of respondents who chose medical staff in our study was lower (16.7% vs 63.2%). The results indicated that stroke health education should be strengthened by medical staff in Chongqing community, in addition to mass media.

There are some limitations in this study. First, the survey was conducted in only three communities in Yuzhong district, so it might not reflect the situation of communities not been selected. Nevertheless, we could generally assess the level of knowledge about thrombolytic therapy for acute ischemic stroke among Chongqing community residents through random sample. Secondly, the response rate was low despite prior notification and publicity in the communities, thus, there was a non-response bias in this cross-sectional survey. The characteristics of the persons who did not participate in the survey were not analyzed in this survey, so we didn’t know if the level of knowledge about thrombolytic therapy for stroke among responding residents differ from those who refused to participate. We speculated several reasons may be related to the low response rate. Some community residents lack awareness of disease prevention and did not believe they would get any benefit from survey, so they might have no enthusiasm of participating in such survey. Some residents might worry about their privacy leakage if participating in such survey. We should further strengthen publicity work before survey, including explaining the purpose and significance of the survey especially the benefit from participating in the survey, and ensuring that all answers of questionnaire were anonymous and participating in this survey would not cause any bad influence. Moreover adding some incentive measures might help to reduce the refusal. Third, compared with the open-ended questionnaire, the closed-ended questionnaire used in our survey might result in higher level of knowledge about thrombolytic therapy, while open-ended question for time window might reduce the recovery rate and efficient of questionnaire, however, the general respondents’ thrombolytic-related knowledge can be reflected by using closed-ended questionnaire.

In summary, this survey showed that level of knowledge about thrombolytic therapy for acute ischemic stroke was very low among the community residents in Chongqing. In order to more effectively reduce pre-hospital delay for stroke patients and increase the thrombolytic therapy rate, it is urgent for future health education to improve the public knowledge about thrombolytic therapy in addition to stroke warning signs.
